# Red dichromatic imaging enhances submucosal visibility during endoscopic submucosal dissection: Pilot study

**DOI:** 10.1055/a-2592-3546

**Published:** 2025-05-16

**Authors:** Tsubasa Ishikawa, Tomoaki Tashima, Tomonori Kawasaki, Kankei Fujimoto, Kei Sugimoto, Takahiro Shin, Takahiro Muramatsu, Yumi Mashimo, Shomei Ryozawa

**Affiliations:** 1 183786 Department of Gastroenterology, Saitama Medical University International Medical Center, Hidaka, Japan; 2 183786 Department of Pathology, Saitama Medical University International Medical Center, Hidaka, Japan

**Keywords:** Endoscopy Upper GI Tract, Endoscopic resection (ESD, EMRc, ...), Endoscopy Lower GI Tract, Endoscopic resection (polypectomy, ESD, EMRc, ...), Quality and logistical aspects, Training

## Abstract

**Background and study aims:**

In 2020, Olympus Medical Systems Corporation introduced red dichromatic imaging (RDI) as a novel image-enhanced endoscopy (IEE) technology. However, clinical evidence regarding its practical applications and the lesions for which RDI is beneficial remains limited. Endoscopic submucosal dissection (ESD) is an essential therapeutic option for gastrointestinal tumors, yet achieving clear visualization of the dissecting layer remains a significant challenge. This study aimed to evaluate the efficacy of RDI in enhancing visualization of the dissecting layer during ESD.

**Methods:**

A total of 86 images from 43 gastrointestinal tumors (esophagus, stomach, duodenum, colon, and rectum) were evaluated by eight endoscopists. Visibility of the dissecting layer was assessed using a scale ranging from -2 (poor) to 2 (excellent) under white light imaging (WLI) and RDI. In addition, the color difference between the submucosal and muscular layers was analyzed using the International Commission on Illumination Lab color space system.

**Results:**

RDI significantly improved visibility of the dissecting layer compared with WLI. Moreover, RDI showed a significantly greater color difference between the submucosal and muscular layers than WLI.

**Conclusions:**

RDI enhances visualization during ESD by improving visibility of the dissecting layer and increasing color differentiation compared with conventional WLI. These findings suggest that incorporating RDI into routine endoscopic practice could lead to more precise and efficient ESD procedures, ultimately improving patient outcomes.

## Introduction

Advancements in endoscopic examination techniques for gastrointestinal tumors have significantly improved optical performance and qualitative diagnostic capabilities in recent years. In particular, widespread use of endoscopic submucosal dissection (ESD), pioneered in Japan, has expanded indications for endoscopic treatment of gastrointestinal tract tumors 1 2 3 4 .

Among these advancements, various optical techniques, including zoom magnification and image-enhanced endoscopy (IEE), are noteworthy and have demonstrated efficacy in enhancing lesion detection rates and improving qualitative diagnosis of gastrointestinal lesions 5 6 7 . Specific technologies such as narrow-band imaging (NBI) 8 9 10 , blue laser imaging (BLI) 11 , and linked color imaging (LCI) 12 have been commonly used in IEE. For instance, NBI utilizes two wavelengths of light (blue light: 390–445 nm; green light: 530–550 nm) to highlight capillaries on the mucosal surface structure, aiding in lesion detection and borderline diagnosis 8 9 10 . Similarly, LCI enhances fluorescence by increasing output of 450-nm light, thereby improving color separation in the red region through image processing 11 12 13 .

Among the latest additions to the armamentarium of IEE technologies are texture and color enhancement imaging (TXI) and red dichromatic imaging (RDI), introduced by Olympus Medical Corporation. TXI is a novel imaging technique that optimizes the "structure," "color tone," and "brightness" of mucosal surfaces in normal white-light imaging (WLI) by separating texture images and base images, performing texture enhancement, color correction, and brightness correction 14 15 .RDI significantly enhances visibility of deep blood vessels and bleeding points using relatively long wavelengths of visible light (520–550, 595–610, and 620–640 nm). Previous studies have reported its effectiveness, particularly in securing the field of view during bleeding situations 16 17 18 19 20 . Given its potential benefits in hemostasis and treatment situations such as ESD, RDI holds promise as a valuable tool in gastrointestinal endoscopy 21 . Although RDI is a newly introduced IEE and its usefulness is currently anticipated, there is little information on the actual clinical situations and lesions for which it is useful 22 23 24 .

In this study, we aimed to clarify efficacy of RDI in patients who underwent ESD for tumors in the upper and lower gastrointestinal tracts.

## Patients and methods

### Instruments

This study utilized the CV-1500 light source equipped with a RDI system in conjunction with PCF-H290Ti, PCF-H209Zi, GIF-H290T, GIF-EZ1500 and GIF-H290Z endoscopes (Olympus Medical Systems Corporation, Tokyo, Japan). RDI offers three modes: mode 1, mode 2, and mode 3, with mode 1 being employed in this study. For the structure-enhanced mode, “A7” was selected for WLI. In the ESD procedure, the following equipment and settings were used: High-frequency knives: TechKnife (Micro-Tech, China) and Dual Knife J (Olympus Medical, Japan), Hemostatic forceps: Coagrasper FD-411QR (Olympus Medical, Japan), Injection needle: Supergrip 25G, 4 mm (TOP, Japan), Injection solution: 1V hyaluronic acid solution (Boston Scientific Japan, Japan) + 0.4% Indigo Carmine solution 1 cc, Endoscopic distal cap: ST Hood (Fujifilm, Japan) and D-201–11804 (Olympus Medical, Japan), Endoscopic clip: EZ Clip (Olympus Medical, Japan), High-frequency generator: VIO3 (ERBE, Germany).

### Overview and features of images

**Fig. 1:**
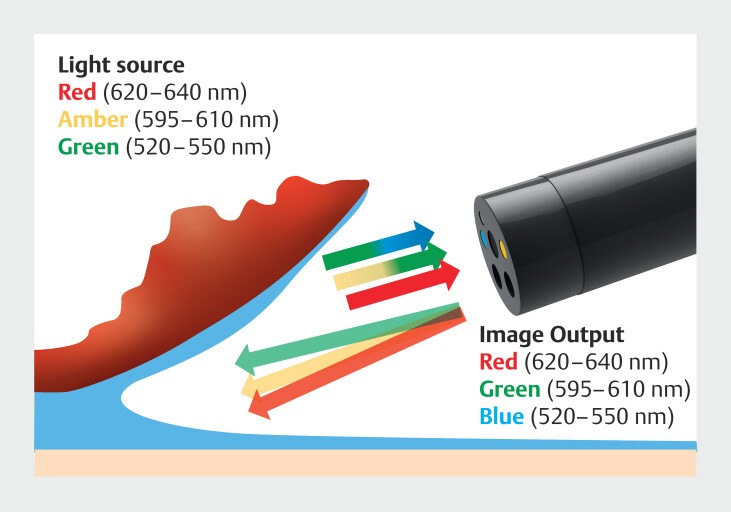
Overview of RDI images. When using RDI, the endoscope emits red (620–640 nm), amber (595–610 nm), and green (520–550 nm) light toward the object. In the endoscopic image, the reflected red light is represented as red, amber light as green, and green light as blue.

The RDI imaging modality functions as follows. RDI illuminates the target with relatively long wavelengths of visible light, specifically within the ranges of 520 to 550 nm (green), 595 to 610 nm (amber), and 620 to 640 nm (red). Reflected light is projected in red (620–640 nm), green (595–610 nm), and blue (520–550 nm) on the screen ( Fig. 1 ). Particularly in hemorrhage scenarios, absorption characteristics of hemoglobin by different wavelengths can accentuate color variations, facilitating identification of bleeding sources (Supplementary Fig. 1) 16 21 .

### Study design and patients

This retrospective study examined patients who underwent ESD for upper and lower gastrointestinal tumors (esophagus, stomach, duodenum, and colorectum) between March 2023 and September 2023 at Saitama Medical University International Medical Center. Only cases in which the submucosa and muscularis mucosae were simultaneously visible during submucosal dissection and images captured during the transition between RDI and WLI were included.

ESD procedures were conducted as part of routine clinical practice, with use of RDI left to the discretion of the attending endoscopist. Cases with comparable compositions between RDI and WLI were selected, whereas lesions with insufficient imaging were excluded. Suitable images for comparison were retrospectively extracted from the videos of included lesions.

Endoscopists then assessed visibility of the submucosa and muscularis mucosae during submucosal dissection, as well as the color difference between these two areas. This study was reviewed and approved by the Institutional Review Board of Saitama Medical University International Medical Center (Approval numbers: 20–249, 22–047, and 20–202). All treatment procedures were performed in accordance with relevant guidelines and regulations.

### Visibility score for submucosal and muscular layers

**Fig. 2:**
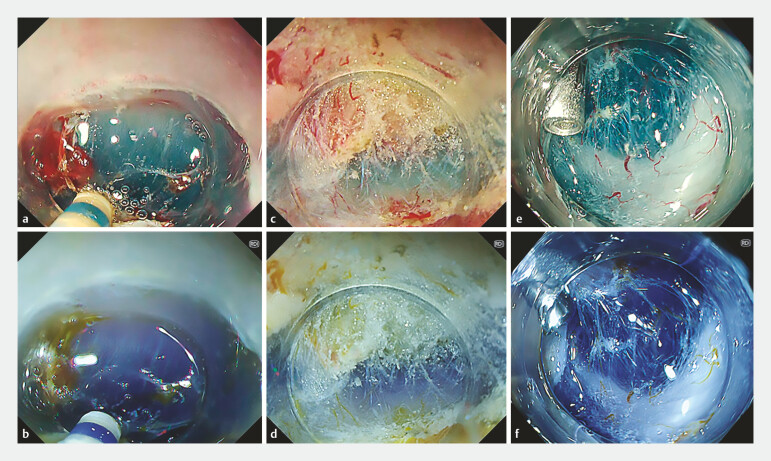
Visibility of dissecting layer during ESD. **a, b** Eophageal ESD. **c, d** Duodenal ESD. **e, f** Colorectal ESD images. WLI images are shown in **a** , **c** , and **e** . RDI images are shown in **b** , **d** , and **f** .

Images were retrospectively extracted from the treatment process video to assess the visibility score for the submucosa and muscle layer. Selection criteria included images in which the submucosa and muscularis were simultaneously visible, the imaging mode was switched between WLI and RDI, and WLI and RDI images were obtained under the same composition ( Fig. 2 ).

**Fig. 3:**
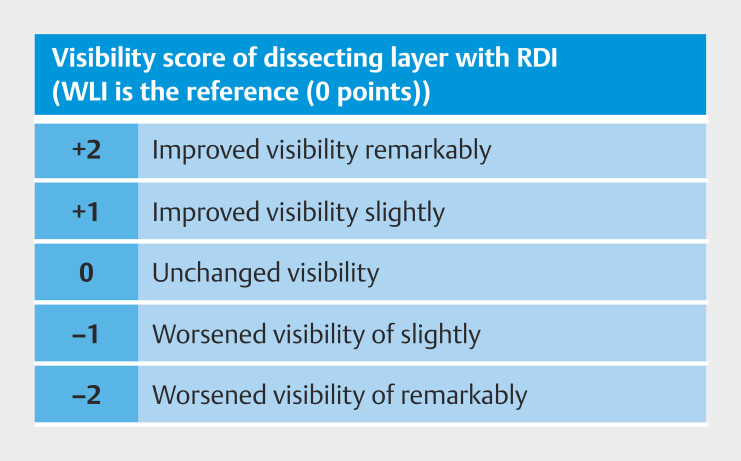
Visibility score of dissecting layers. For assessing visibility of the dissecting layer in ESD, visibility score by RDI was determined relative to WLI (with WLI scoring 0 points) as follows: +2: Visibility significantly improved, +1: Visibility slightly improved, 0: Visibility comparable to WLI, –1: Visibility slightly worse, –2: Visibility significantly worse.

Eight endoscopists, including four experts and four trainees, evaluated visibility of the extracted WLI and RDI images. Experts, defined as endoscopists with at least 5 years of IEE experience, were compared with trainees, who were residents with less than 5 years of IEE experience. Images were electronically displayed without zooming, ensuring consistency. All images were standardized to the same size and reviewed on a routinely used endoscopy monitor. Subsequently, they were assessed based on a visibility scoring method ( Fig. 3 ). The scoring system categorized visibility by WLI as the baseline (=0), whereas visibility by RDI was evaluated on a 5-point scale: 2 for excellent (easily distinguished compared to WLI), 1 for good (somewhat easily distinguished compared to WLI), 0 for normal (similar to WLI), -1 for fair (slightly more difficult to distinguish than WLI), and -2 for poor (clearly more difficult to distinguish than WLI).

### Color difference between the submucosa and the muscular layer

**Fig. 4:**
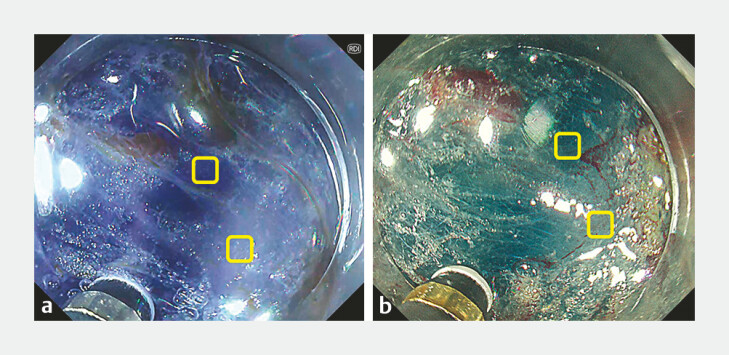
Examples of color sampling in calculation of color difference. Colors were sampled from the submucosa and muscle layer to determine the dissecting layer during ESD. Samples were collected in the same manner from both the submucosa and muscle layers, and color differences were calculated using the CIELAB color space system in Adobe Photoshop. a Image taken with RDI, with the areas sampled from the submucosa and nearby muscle layers indicated by yellow boxes. b Image taken with WLI, highlighting the sampled areas from the submucosa and nearby muscle layers.

Color difference (ΔE) was determined using the International Commission on Illumination L*a*b* (CIELAB) color space system 25 . The CIELAB color space constitutes a three-dimensional model consisting of a black-white axis (L*, lightness), a red-green axis (a*, red-green component), and a yellow-blue axis (b*, yellow-blue component), devised to approximate human perception. The Euclidean distance between two points in the CIELAB color space is directly proportional to disparity in color perception. ΔE values, calculated according to the CIELAB color space 25 26 , were used to evaluate the perceived color difference in endoscopic images during ESD submucosal dissection; the color difference between the submucosa and the muscular layer was evaluated by comparing the WLI image with an RDI image of identical composition ( Fig. 4 ). The color of the submucosa and the muscularis layer was sampled from 31 × 31 pixels of the 567 × 526-pixel image, and the average value was used in the study. In addition, colors were sampled from the corresponding locations in WLI and RDI. Color values of the sampled sites were evaluated and scored based on the L* a* b* color values of the CIELAB color space system using Adobe Photoshop CC 2023, as previously reported 26 27 . To calculate color difference, the following equation was used: ΔE* = [(ΔL*) ^2^ + (Δa*) ^2^ + (Δb*) ^2^ ] ^1/2^ . Furthermore, pixels affected by halation were excluded.

### Statistical analysis

Clinical data were expressed as percentages, means, and standard deviations (SDs). The mean and SD of the visibility score and color difference (ΔE) were calculated. RDI visibility scores, evaluated by all endoscopists (experts and trainees), were also analyzed for each endoscopist in comparison with the WLI. Scores across modalities were compared using a paired T-test with correspondence. In addition, *P* < 0.05 indicated statistical significance. All statistical data were analyzed using SPSS v.29.0 for Windows (IBM Japan, Tokyo, Japan).

## Results

### Patients and lesions

Overall, 43 patients, comprising 26 males and 17 females with a mean age of 68.1 ± 11.4 years, were included in the analysis, along with 43 lesions. Lesions were distributed across the stomach (16.3%), esophagus (14.0%), duodenum (11.6%), and colorectum (58.1%). Mean lesion diameter (mean ± SD) was 33.8 ± 18.5 mm. Histopathological analysis revealed squamous cell carcinoma in five cases (11.6%), adenocarcinoma in 31 cases (72.1%), and adenoma in seven cases (16.3%). Submucosal fibrosis was observed in 27 cases (62.8%) ( Table 1 ).

**Table:** **Table 1** Patient and lesion characteristics.

	n = 43
Age (mean ± SD)	68.1 ± 11.4
Sex (male/female)	26/17
Tumor size (mean ± SD, mm)	33.8 ± 18.5
Site of the lesion, n (%)	
Esophagus	7 (16.3)
Stomach	6 (14.0)
Duodenum	5 (11.6)
Colorectum	25 (58.1)
Histopathological diagnosis, n (%)	
Adenoma	7 (16.3)
Adenocarcinoma	31 (72.1)
Squamous cell carcinoma	5 (11.6)
Fibrosis of the submucosal layer, n(%)	
F0	16 (37.2)
F1	13 (30.2)
F2	14 (32.6)
F0, without fibrosis; F1, slight fibrosis; F2, severe fibrosis; SD, standard deviation	

### Visibility score for submucosal and muscular layers

Among the six endoscopists who participated in the evaluation, RDI demonstrated superior visibility for the dissecting layer (submucosa) compared with WLI (with WLI assigned a reference score of 0, *P* < 0.01). Table 2 summarizes the visibility score results, including assessments by experts and trainees.

**Table:** **Table 2** Mean visibility scores of gastric neoplasms for WLI, TXI mode 1, TXI mode 2 indigo-WLI, indigo-TXI mode 1, and indigo-TXI mode 2.

	Visibility score of dissecting layers (mean ± SD)	*P* value
	(RDI vs. WLI [score 0])	
Expert A	1.72 ± 0.45	< 0.0001
Expert B	1.63 ± 0.61	< 0.0001
Expert C	1.16 ± 0.57	< 0.0001
Expert D	1.27 ± 0.62	< 0.0001
Trainee E	1.51 ± 0.54	< 0.0001
Trainee F	1.44 ± 0.73	< 0.0001
Trainee G	1.63 ± 0.53	< 0.0001
Trainee H	1.65 ± 0.52	< 0.0001
RDI, red dichromatic imaging; SD, standard deviation; WLI, white-light imaging.		

### Color difference between submucosa and muscular layer

The color difference (ΔE) between the muscular and submucosal layers was significantly higher in RDI mode than in WLI (17.2 ± 9.7 vs. 23.7 ± 9.1, *P* = 0.002), as detailed in Table 3 . In addition, the color difference in L value (brightness) alone in the LAB color space system was calculated to reflect the improvement in transparency due to RDI, summarized in Table 4 (ΔE-L). ΔE-L was also significantly higher in RDI mode than in WLI mode (8.5 ± 6.1 vs. 16.4 ± 6.0, *P* = 0.001).

**Table:** **Table 3** Objective evaluations using color differences (ΔE*; mean ± SD) between submucosa and muscle layer when using WLI and RDI according to L* a* b* values.

	L* a* b* values	WLI	RDI	*P* value
Muscle layer vs. submucosal layer	ΔE*	17.2 ± 9.7	23.7 ± 9.1	0.002*
RDI, red dichromatic imaging; SD, standard deviation; WLI, white-light imaging. *Paired T-test.				

**Table:** **Table 4** Objective evaluations using color differences (ΔE*; mean ± SD) between submucosa and muscularis layer when using WLI and RDI by L* values.

	L* values	WLI	RDI	*P* value
Muscle layer vs. submucosal layer	ΔE-L*	8.5 ± 6.1	16.4 ± 6.0	0.001*
RDI, red dichromatic imaging; SD, standard deviation; WLI, white-light imaging. *Paired T-test.				

## Discussion

In ESD procedures, visualizing the submucosa is crucial for accurate dissection while distinguishing it from the muscular layer. Recognizing the presence of the muscle layer behind the submucosa is important, and RDI holds promise in enhancing this visualization. RDI utilizes a long wavelength light source, which is expected to minimize scattering and penetration into the tissue (Supplementary Fig. 1) 19 20 28 . Therefore, our study focused on comparing visibility and color differentiation between the submucosa and muscular layer using RDI and WLI, both commonly used in ESD procedures, employing the CIELAB color space system.

We will first delve into the fundamental properties of RDI. RDI employs red, amber, and green as irradiation light sources. On the endoscope screen, the reflected light from green is depicted as blue, from amber as green, and from red as red ( Fig. 1 ). During hemorrhage, amber is believed to have higher absorbance than red due to abrupt changes in absorption scattering around the 600-nm wavelength 17 .

Consequently, output is perceived as a combination of dark red and light green in areas of darker hemoglobin concentration, creating discernible differences between darker and lighter hemorrhage spots 17 21 . In practical terms, RDI yields clearer images, possibly due to its emission of higher-wavelength photons, which are less prone to scattering and may be less affected by small particles.

In ESD, the submucosa is typically injected with an indigo carmine solution. During submucosal dissection, submucosa containing indigo carmine absorbs red as wavelengths but retains green irradiation information. This phenomenon likely contributes to the more vivid blue appearance of the submucosa when RDI is utilized in ESD.

Moreover, red irradiation light is easily absorbed but not scattered, facilitating visualization of the muscle layer behind the submucosa, particularly when the muscle layer exists behind the submucosa. Because all wavelengths of red, amber, and green light are reflected in the muscle layer, it may appear to be a white tone on the endoscope screen (Supplementary Fig. 1).

The results of this study demonstrate that ESD using RDI significantly enhances the distinction between the muscular and submucosal layers, compared with WLI, which is a crucial aspect during dissection operations. In addition, the color difference (ΔE) between the submucosa and the muscularis layer is significantly higher with RDI than with WLI.

In clinical practice, visualizing the submucosa with RDI suggests enhanced depth perception compared with WLI, possibly due to reduced scatter resulting from the longer wavelength. However, it is hypothesized that the perceived high transparency with RDI may be attributable to less reflected light information, resulting in a darker endoscopic image 19 20 21 28 . Therefore, we investigated the color difference in brightness (ΔE-L), revealing a significantly higher difference with RDI compared to WLI.

These results suggest the potential utility of RDI in improving visibility of the dissecting submucosal layer during ESD procedures. Further data accumulation, including randomized trials at multiple centers, is warranted to validate these results and explore additional applications in the future.

However, this study has a few limitations. First, it was small, single-center, and retrospective. Second, the number of images evaluated was limited. Third, visibility was assessed using still images rather than video. Normally, visibility during treatment and the ease of dissection should be assessed through video. However, in this study, it was impossible to perform the same procedure in the same scenario using RDI and WLI. Therefore, the mode switch could only be conducted at a single point when the submucosal layer was visible. Future research will include not only still images but also videos for a more thorough evaluation. Fourth, endoscopists had a predisposition to expect better visibility with the RDI mode due to its inherent characteristics. Larger sample sizes are warranted for future studies, and clinical outcomes, such as dissection speed, should be examined in a multicenter, prospective, randomized controlled trial (RCT). Fifth, regarding image identity, RDI and WLI images were not derived from the exact same WLI image; although the same composition and subject were consecutively captured in RDI and WLI, they were obtained at different times. Hence, this aspect may pose a limitation. Sixth, the study did not examine the concentration of indigo carmine dye used in the local injection solution. In this research, we uniformly used a hyaluronic acid solution with the indigo carmine solution as described in the Methods section, but dye concentration was not assessed. Future investigations should consider an appropriate concentration of indigo carmine. Seventh, this study did not evaluate clinical outcomes such as dissection speed, en bloc resection rate, and incidence of complications.

Eighth, timing of RDI usage during ESD was at operator discretion. However, in many cases, most of the incision and dissection procedures were performed using RDI, which may introduce selection bias. Ninth, color values used for color difference calculations were sampled from regions identified by the authors as submucosal and adjacent muscular layers, which may not be entirely accurate.

In the future, multicenter studies, including RCTs that limit the treatment process to those using RDI or WLI, will be necessary. It is anticipated that RDI, which facilitates differentiation between the muscular layer and submucosal layer, will lead to improvements in dissection speed.

## Conclusions

RDI is considered useful for distinguishing the submucosal layer from other layers during ESD, making it a valuable imaging modality for this procedure.

## Supplementary Material

Supplementary Material Zusatzmaterial
