# Supplementary material for: Red dichromatic imaging enhances submucosal visibility during endoscopic submucosal dissection: Pilot study
Source: Endosc Int Open. 2025 May 16;13:a25923546. doi: 10.1055/a-2592-3546 (PMC12090977; doi:10.1055/a-2592-3546)

Supplementary Fig. 1

A : Absorption properties of RDI-used wavelengths for hemoglobin (Hb). The absorption characteristics of Hb are shown: the Green and Amber irradiation light used for RDI have strong absorption for Hb, while the Red wavelength band has low absorption. This is believed to be useful for visualization of bleeding points.

B : Transmittance at different wavelengths to human gastric mucosa. The long wavelength band, which is the main component in RDI, is more transparent than the short wavelength band.

C : Absorption rate of various wavelengths to human gastric mucosa. The long wavelength band, the main component of RDI, has lower absorption than the short wavelength band.

D : Differences in reflectance in human gastric mucosa according to wavelength. The long wavelength band, which is the main component of RDI, shows higher reflectance than the lower wavelength band.

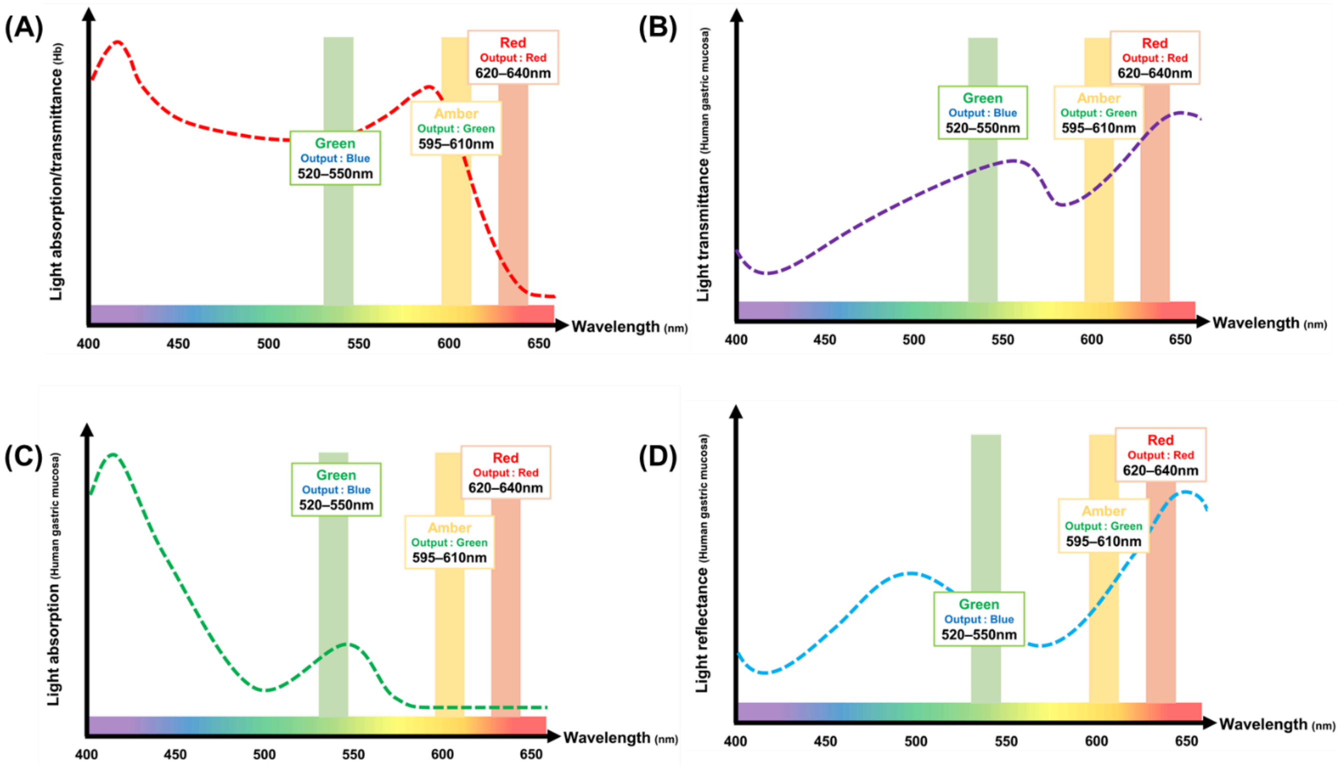

Supplement: Supplementary file 1 — Supplementary Material [file 10-1055-a-2592-3546_25937832.pdf]
